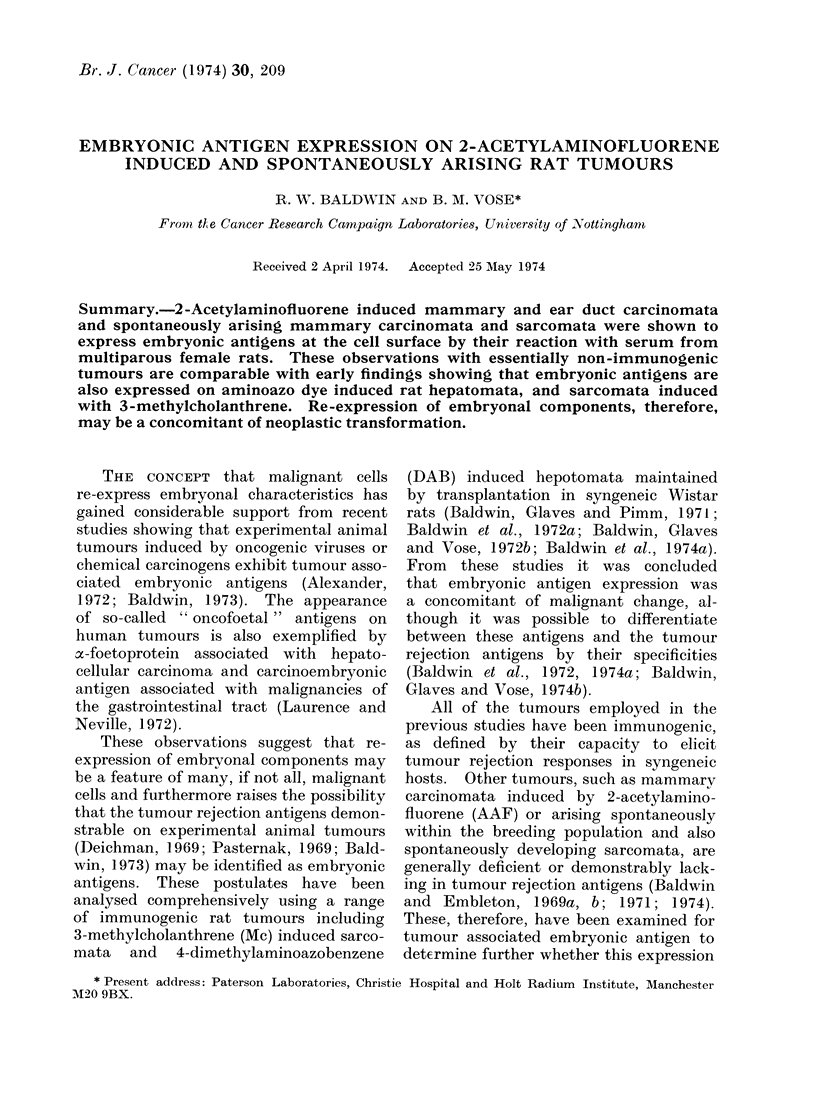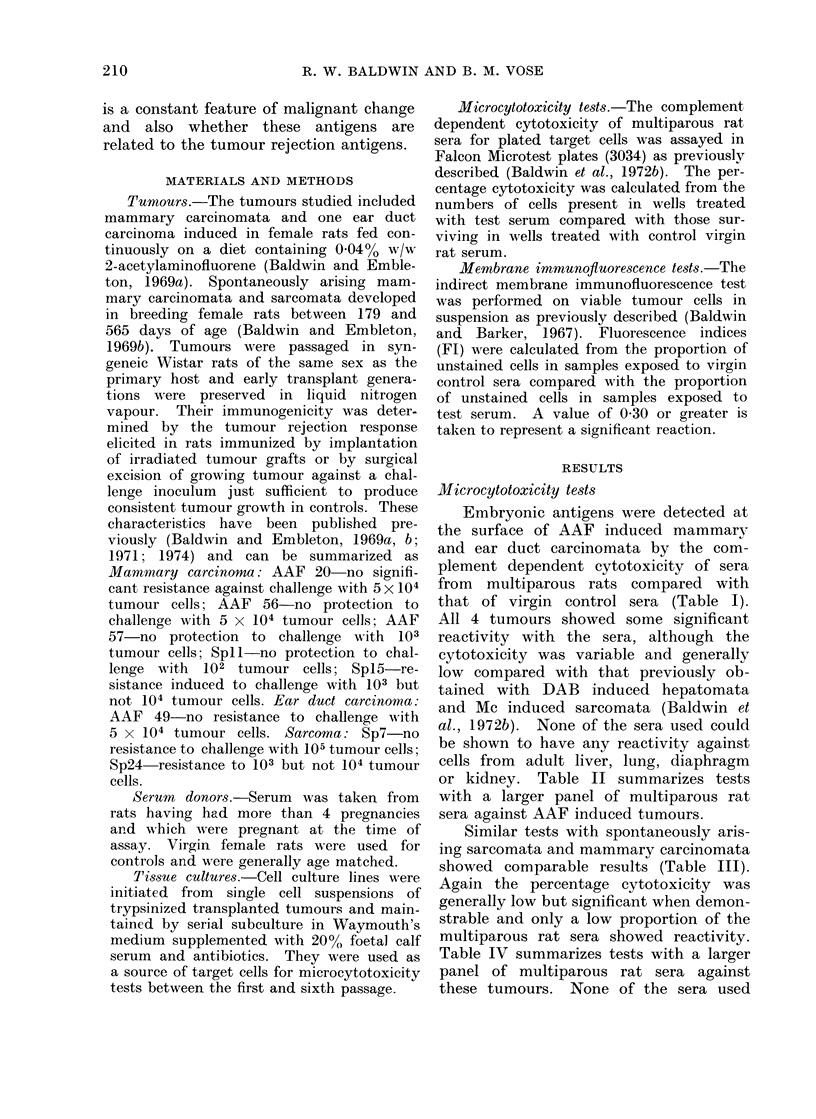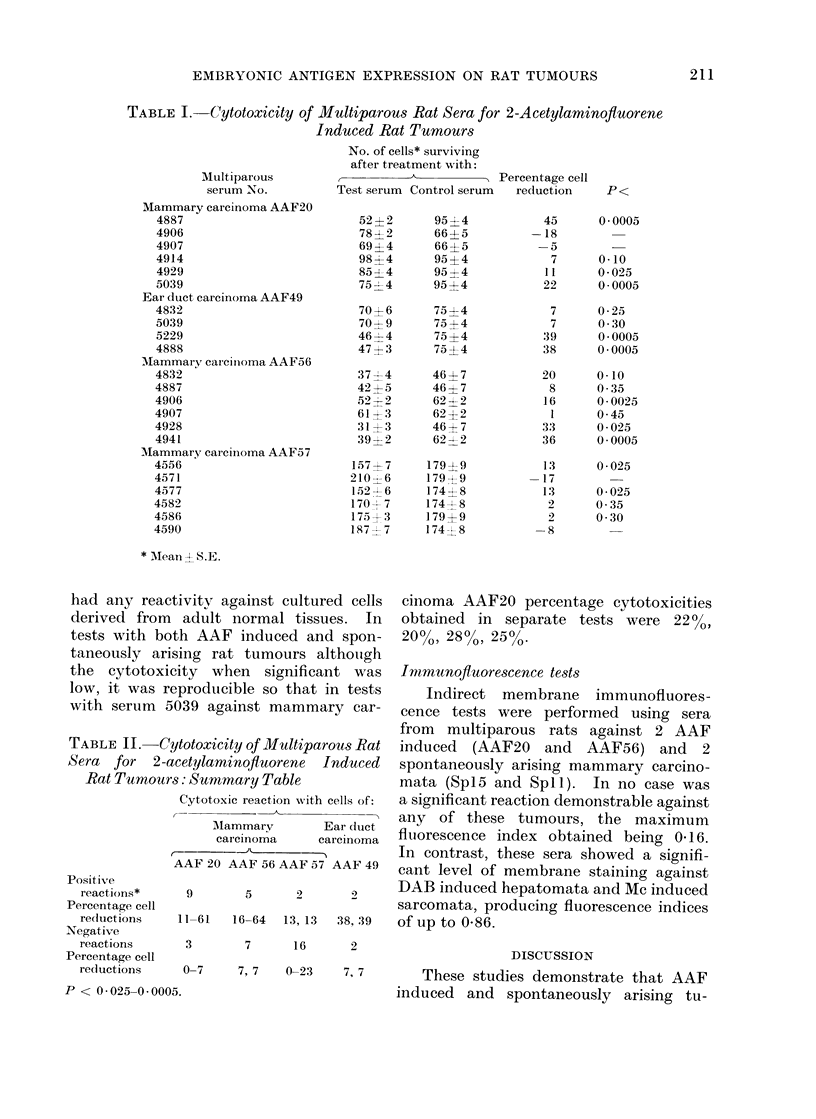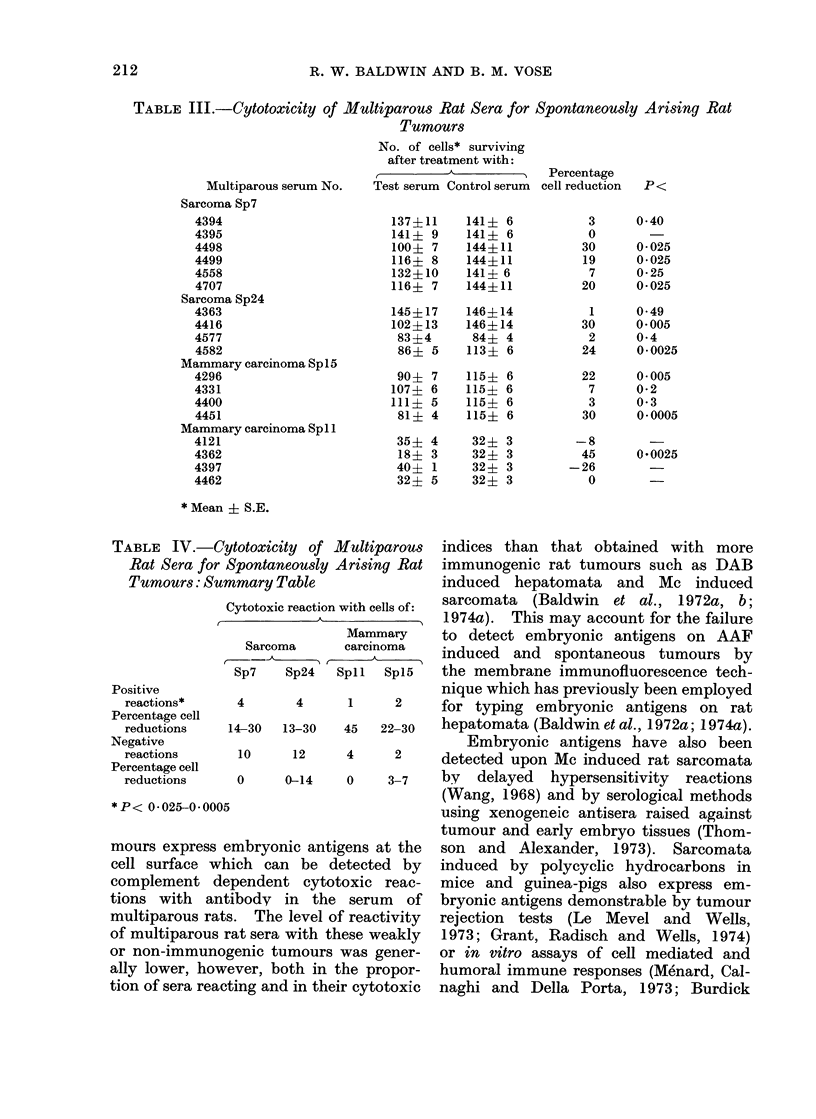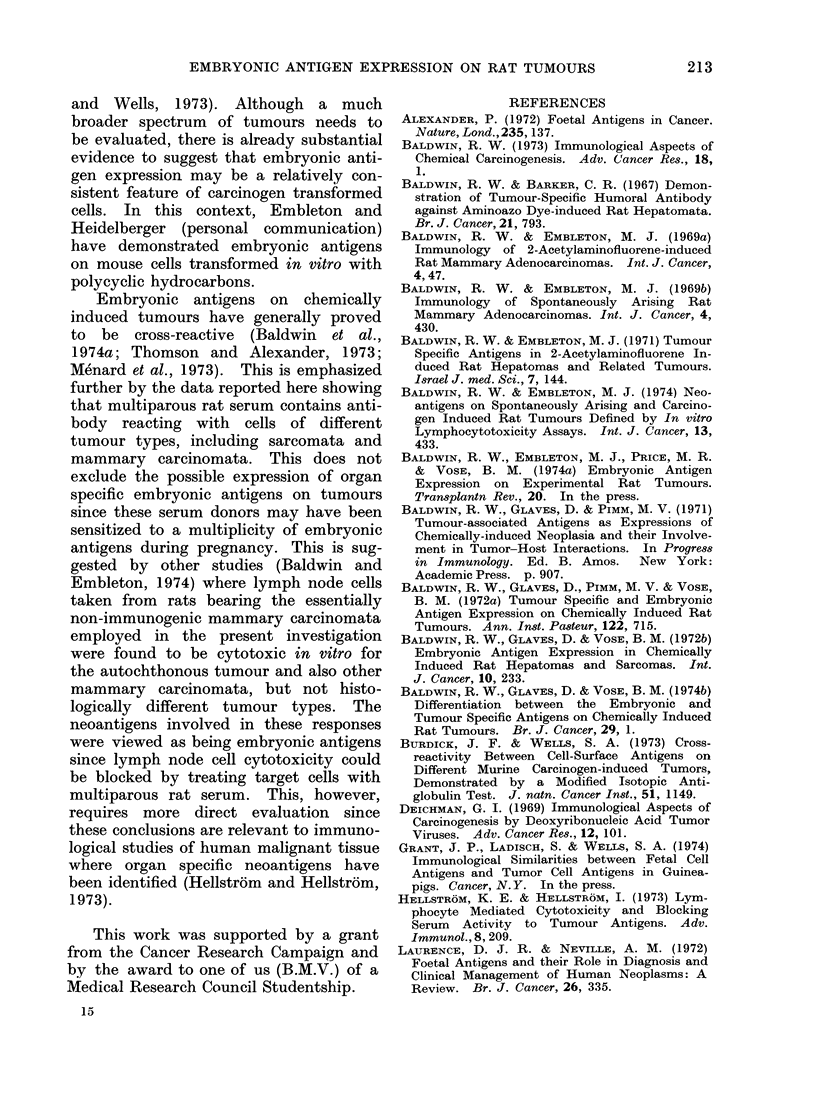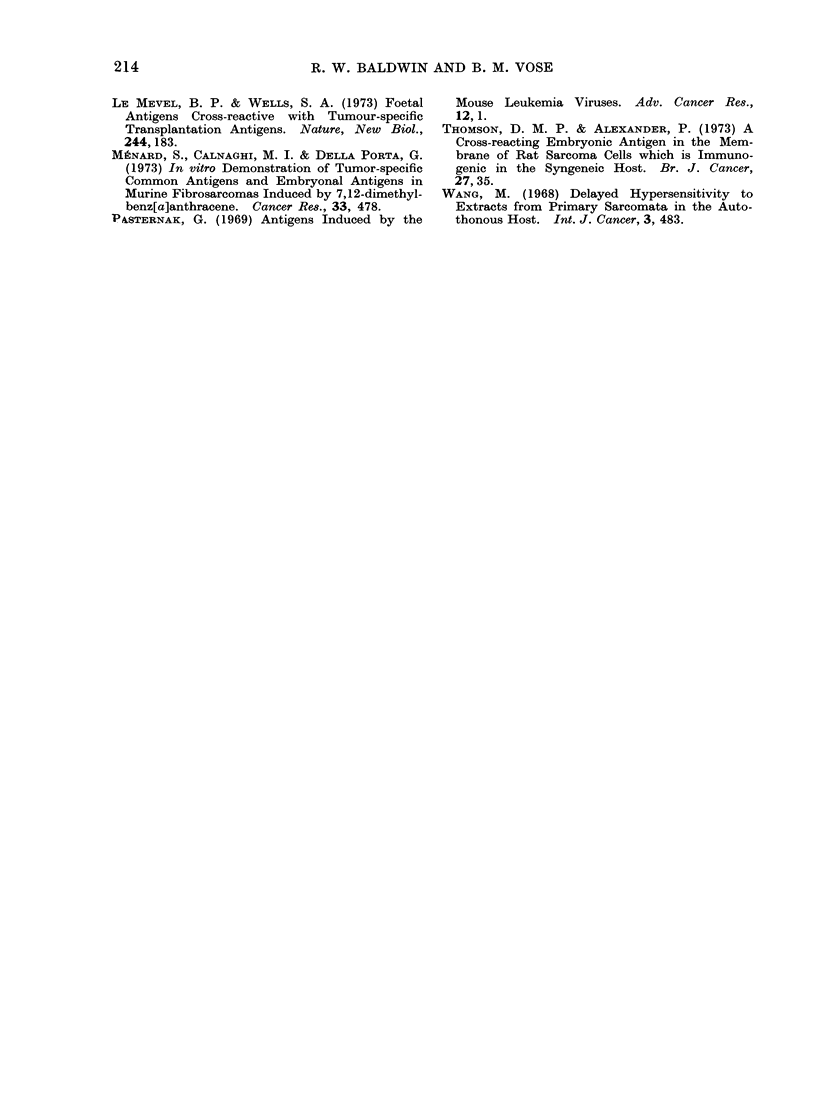# Embryonic Antigen Expression on 2-Acetylaminofluorene Induced and Spontaneously Arising Rat Tumours

**DOI:** 10.1038/bjc.1974.183

**Published:** 1974-09

**Authors:** R. W. Baldwin, B. M. Vose

## Abstract

2-Acetylaminofluorene induced mammary and ear duct carcinomata and spontaneously arising mammary carcinomata and sarcomata were shown to express embryonic antigens at the cell surface by their reaction with serum from multiparous female rats. These observations with essentially non-immunogenic tumours are comparable with early findings showing that embryonic antigens are also expressed on aminoazo dye induced rat hepatomata, and sarcomata induced with 3-methylcholanthrene. Re-expression of embryonal components, therefore, may be a concomitant of neoplastic transformation.


*Br. J. Cancer* (1974) **30**, 209

### EMBRYONIC ANTIGEN EXPRESSION ON 2-ACETYLAMINOFLUORENE INDUCED AND SPONTANEOUSLY ARISING RAT TUMOURS

R. W. BALDWIN AND B. M. VOSE*

*From the Cancer Research Campaign Laboratories, University of Nottingham*

Received 2 April 1974. Accepted 25 May 1974

**Summary.—2-Acetylaminofluorene induced mammary and ear duct carcinomata and spontaneously arising mammary carcinomata and sarcomata were shown to express embryonic antigens at the cell surface by their reaction with serum from multiparous female rats. These observations with essentially non-immunogenic tumours are comparable with early findings showing that embryonic antigens are also expressed on aminoazo dye induced rat hepatomata, and sarcomata induced with 3-methylcholanthrene. Re-expression of embryonal components, therefore, may be a concomitant of neoplastic transformation.**

THE CONCEPT that malignant cells re-express embryonal characteristics has gained considerable support from recent studies showing that experimental animal tumours induced by oncogenic viruses or chemical carcinogens exhibit tumour associated embryonic antigens (Alexander, 1972; Baldwin, 1973). The appearance of so-called "oncofoetal" antigens on human tumours is also exemplified by α-foetoprotein associated with hepatocellular carcinoma and carcinoembryonic antigen associated with malignancies of the gastrointestinal tract (Laurence and Neville, 1972).

These observations suggest that re-expression of embryonal components may be a feature of many, if not all, malignant cells and furthermore raises the possibility that the tumour rejection antigens demonstrable on experimental animal tumours (Deichman, 1969; Pasternak, 1969; Baldwin, 1973) may be identified as embryonic antigens. These postulates have been analysed comprehensively using a range of immunogenic rat tumours including 3-methylcholanthrene (Mc) induced sarcomata and 4-dimethylaminoazobenzene (DAB) induced hepotomata maintained by transplantation in syngeneic Wistar rats (Baldwin, Glaves and Pimm, 1971; Baldwin *et al.*, 1972*a*; Baldwin, Glaves and Vose, 1972*b*; Baldwin *et al.*, 1974*a*). From these studies it was concluded that embryonic antigen expression was a concomitant of malignant change, although it was possible to differentiate between these antigens and the tumour rejection antigens by their specificities (Baldwin *et al.*, 1972, 1974*a*; Baldwin, Glaves and Vose, 1974*b*).

All of the tumours employed in the previous studies have been immunogenic, as defined by their capacity to elicit tumour rejection responses in syngeneic hosts. Other tumours, such as mammary carcinomata induced by 2-acetylaminofluorene (AAF) or arising spontaneously within the breeding population and also spontaneously developing sarcomata, are generally deficient or demonstrably lacking in tumour rejection antigens (Baldwin and Embleton, 1969*a*, *b*; 1971; 1974). These, therefore, have been examined for tumour associated embryonic antigen to determine further whether this expression

* Present address: Paterson Laboratories, Christie Hospital and Holt Radium Institute, Manchester M20 9BX.

is a constant feature of malignant change and also whether these antigens are related to the tumour rejection antigens.

### MATERIALS AND METHODS

*Tumours.*—The tumours studied included mammary carcinomata and one ear duct carcinoma induced in female rats fed continuously on a diet containing 0·04% w/w 2-acetylaminofluorene (Baldwin and Embleton, 1969*a*). Spontaneously arising mammary carcinomata and sarcomata developed in breeding female rats between 179 and 565 days of age (Baldwin and Embleton, 1969*b*). Tumours were passaged in syngeneic Wistar rats of the same sex as the primary host and early transplant generations were preserved in liquid nitrogen vapour. Their immunogenicity was determined by the tumour rejection response elicited in rats immunized by implantation of irradiated tumour grafts or by surgical excision of growing tumour against a challenge inoculum just sufficient to produce consistent tumour growth in controls. These characteristics have been published previously (Baldwin and Embleton, 1969*a*, *b*; 1971; 1974) and can be summarized as *Mammary carcinoma:* AAF 20—no significant resistance against challenge with $5 \times 10^4$ tumour cells; AAF 56—no protection to challenge with $5 \times 10^4$ tumour cells; AAF 57—no protection to challenge with $10^3$ tumour cells; Sp11—no protection to challenge with $10^2$ tumour cells; Sp15—resistance induced to challenge with $10^3$ but not $10^4$ tumour cells. *Ear duct carcinoma:* AAF 49—no resistance to challenge with $5 \times 10^4$ tumour cells. *Sarcoma:* Sp7—no resistance to challenge with $10^5$ tumour cells; Sp24—resistance to $10^3$ but not $10^4$ tumour cells.

*Serum donors.*—Serum was taken from rats having had more than 4 pregnancies and which were pregnant at the time of assay. Virgin female rats were used for controls and were generally age matched.

*Tissue cultures.*—Cell culture lines were initiated from single cell suspensions of trypsinized transplanted tumours and maintained by serial subculture in Waymouth's medium supplemented with 20% foetal calf serum and antibiotics. They were used as a source of target cells for microcytotoxicity tests between the first and sixth passage.

*Microcytotoxicity tests.*—The complement dependent cytotoxicity of multiparous rat sera for plated target cells was assayed in Falcon Microtest plates (3034) as previously described (Baldwin *et al.*, 1972*b*). The percentage cytotoxicity was calculated from the numbers of cells present in wells treated with test serum compared with those surviving in wells treated with control virgin rat serum.

*Membrane immunofluorescence tests.*—The indirect membrane immunofluorescence test was performed on viable tumour cells in suspension as previously described (Baldwin and Barker, 1967). Fluorescence indices (FI) were calculated from the proportion of unstained cells in samples exposed to virgin control sera compared with the proportion of unstained cells in samples exposed to test serum. A value of 0·30 or greater is taken to represent a significant reaction.

### RESULTS

### *Microcytotoxicity tests*

Embryonic antigens were detected at the surface of AAF induced mammary and ear duct carcinomata by the complement dependent cytotoxicity of sera from multiparous rats compared with that of virgin control sera (Table I). All 4 tumours showed some significant reactivity with the sera, although the cytotoxicity was variable and generally low compared with that previously obtained with DAB induced hepatomata and Mc induced sarcomata (Baldwin *et al.*, 1972*b*). None of the sera used could be shown to have any reactivity against cells from adult liver, lung, diaphragm or kidney. Table II summarizes tests with a larger panel of multiparous rat sera against AAF induced tumours.

Similar tests with spontaneously arising sarcomata and mammary carcinomata showed comparable results (Table III). Again the percentage cytotoxicity was generally low but significant when demonstrable and only a low proportion of the multiparous rat sera showed reactivity. Table IV summarizes tests with a larger panel of multiparous rat sera against these tumours. None of the sera used

TABLE I.—*Cytotoxicity of Multiparous Rat Sera for 2-Acetylaminofluorene Induced Rat Tumours*

| Multiparous serum No. | No. of cells* surviving after treatment with: Test serum | Control serum | Percentage cell reduction | $P <$ |
|---|---|---|---|---|
| Mammary carcinoma AAF20 | | | | |
| 4887 | 52±2 | 95±4 | 45 | 0·0005 |
| 4906 | 78±2 | 66±5 | −18 | — |
| 4907 | 69±4 | 66±5 | −5 | — |
| 4914 | 98±4 | 95±4 | 7 | 0·10 |
| 4929 | 85±4 | 95±4 | 11 | 0·025 |
| 5039 | 75±4 | 95±4 | 22 | 0·0005 |
| Ear duct carcinoma AAF49 | | | | |
| 4832 | 70±6 | 75±4 | 7 | 0·25 |
| 5039 | 70±9 | 75±4 | 7 | 0·30 |
| 5229 | 46±4 | 75±4 | 39 | 0·0005 |
| 4888 | 47±3 | 75±4 | 38 | 0·0005 |
| Mammary carcinoma AAF56 | | | | |
| 4832 | 37±4 | 46±7 | 20 | 0·10 |
| 4887 | 42±5 | 46±7 | 8 | 0·35 |
| 4906 | 52±2 | 62±2 | 16 | 0·0025 |
| 4907 | 61±3 | 62±2 | 1 | 0·45 |
| 4928 | 31±3 | 46±7 | 33 | 0·025 |
| 4941 | 39±2 | 62±2 | 36 | 0·0005 |
| Mammary carcinoma AAF57 | | | | |
| 4556 | 157±7 | 179±9 | 13 | 0·025 |
| 4571 | 210±6 | 179±9 | −17 | — |
| 4577 | 152±6 | 174±8 | 13 | 0·025 |
| 4582 | 170±7 | 174±8 | 2 | 0·35 |
| 4586 | 175±3 | 179±9 | 2 | 0·30 |
| 4590 | 187±7 | 174±8 | −8 | — |

* Mean ± S.E.

had any reactivity against cultured cells derived from adult normal tissues. In tests with both AAF induced and spontaneously arising rat tumours although the cytotoxicity when significant was low, it was reproducible so that in tests with serum 5039 against mammary carcinoma AAF20 percentage cytotoxicities obtained in separate tests were 22%, 20%, 28%, 25%.

TABLE II.—*Cytotoxicity of Multiparous Rat Sera for 2-acetylaminofluorene Induced Rat Tumours: Summary Table*

| | Cytotoxic reaction with cells of: Mammary carcinoma AAF 20 | AAF 56 | AAF 57 | Ear duct carcinoma AAF 49 |
|---|---|---|---|---|
| Positive reactions* | 9 | 5 | 2 | 2 |
| Percentage cell reductions | 11–61 | 16–64 | 13, 13 | 38, 39 |
| Negative reactions | 3 | 7 | 16 | 2 |
| Percentage cell reductions | 0–7 | 7, 7 | 0–23 | 7, 7 |

* $P < 0{\cdot}025–0{\cdot}0005$.

*Immunofluorescence tests*

Indirect membrane immunofluorescence tests were performed using sera from multiparous rats against 2 AAF induced (AAF20 and AAF56) and 2 spontaneously arising mammary carcinomata (Sp15 and Sp11). In no case was a significant reaction demonstrable against any of these tumours, the maximum fluorescence index obtained being 0·16. In contrast, these sera showed a significant level of membrane staining against DAB induced hepatomata and Mc induced sarcomata, producing fluorescence indices of up to 0·86.

### DISCUSSION

These studies demonstrate that AAF induced and spontaneously arising tu-

TABLE III.—*Cytotoxicity of Multiparous Rat Sera for Spontaneously Arising Rat Tumours*

| Multiparous serum No. | No. of cells* surviving after treatment with: Test serum | Control serum | Percentage cell reduction | $P<$ |
|---|---|---|---|---|
| Sarcoma Sp7 | | | | |
| 4394 | 137±11 | 141± 6 | 3 | 0·40 |
| 4395 | 141± 9 | 141± 6 | 0 | — |
| 4498 | 100± 7 | 144±11 | 30 | 0·025 |
| 4499 | 116± 8 | 144±11 | 19 | 0·025 |
| 4558 | 132±10 | 141± 6 | 7 | 0·25 |
| 4707 | 116± 7 | 144±11 | 20 | 0·025 |
| Sarcoma Sp24 | | | | |
| 4363 | 145±17 | 146±14 | 1 | 0·49 |
| 4416 | 102±13 | 146±14 | 30 | 0·005 |
| 4577 | 83±4 | 84± 4 | 2 | 0·4 |
| 4582 | 86± 5 | 113± 6 | 24 | 0·0025 |
| Mammary carcinoma Sp15 | | | | |
| 4296 | 90± 7 | 115± 6 | 22 | 0·005 |
| 4331 | 107± 6 | 115± 6 | 7 | 0·2 |
| 4400 | 111± 5 | 115± 6 | 3 | 0·3 |
| 4451 | 81± 4 | 115± 6 | 30 | 0·0005 |
| Mammary carcinoma Sp11 | | | | |
| 4121 | 35± 4 | 32± 3 | −8 | — |
| 4362 | 18± 3 | 32± 3 | 45 | 0·0025 |
| 4397 | 40± 1 | 32± 3 | −26 | — |
| 4462 | 32± 5 | 32± 3 | 0 | — |

* Mean ± S.E.

TABLE IV.—*Cytotoxicity of Multiparous Rat Sera for Spontaneously Arising Rat Tumours: Summary Table*

| | Cytotoxic reaction with cells of: Sarcoma | | Mammary carcinoma | |
|---|---|---|---|---|
| | Sp7 | Sp24 | Sp11 | Sp15 |
| Positive reactions* | 4 | 4 | 1 | 2 |
| Percentage cell reductions | 14–30 | 13–30 | 45 | 22–30 |
| Negative reactions | 10 | 12 | 4 | 2 |
| Percentage cell reductions | 0 | 0–14 | 0 | 3–7 |

* $P<$ 0·025–0·0005

mours express embryonic antigens at the cell surface which can be detected by complement dependent cytotoxic reactions with antibody in the serum of multiparous rats. The level of reactivity of multiparous rat sera with these weakly or non-immunogenic tumours was generally lower, however, both in the proportion of sera reacting and in their cytotoxic indices than that obtained with more immunogenic rat tumours such as DAB induced hepatomata and Mc induced sarcomata (Baldwin *et al.*, 1972*a*, *b*; 1974*a*). This may account for the failure to detect embryonic antigens on AAF induced and spontaneous tumours by the membrane immunofluorescence technique which has previously been employed for typing embryonic antigens on rat hepatomata (Baldwin *et al.*, 1972*a*; 1974*a*).

Embryonic antigens have also been detected upon Mc induced rat sarcomata by delayed hypersensitivity reactions (Wang, 1968) and by serological methods using xenogeneic antisera raised against tumour and early embryo tissues (Thomson and Alexander, 1973). Sarcomata induced by polycyclic hydrocarbons in mice and guinea-pigs also express embryonic antigens demonstrable by tumour rejection tests (Le Mevel and Wells, 1973; Grant, Radisch and Wells, 1974) or *in vitro* assays of cell mediated and humoral immune responses (Ménard, Calnaghi and Della Porta, 1973; Burdick and Wells, 1973). Although a much broader spectrum of tumours needs to be evaluated, there is already substantial evidence to suggest that embryonic antigen expression may be a relatively consistent feature of carcinogen transformed cells. In this context, Embleton and Heidelberger (personal communication) have demonstrated embryonic antigens on mouse cells transformed *in vitro* with polycyclic hydrocarbons.

Embryonic antigens on chemically induced tumours have generally proved to be cross-reactive (Baldwin *et al.*, 1974*a*; Thomson and Alexander, 1973; Ménard *et al.*, 1973). This is emphasized further by the data reported here showing that multiparous rat serum contains antibody reacting with cells of different tumour types, including sarcomata and mammary carcinomata. This does not exclude the possible expression of organ specific embryonic antigens on tumours since these serum donors may have been sensitized to a multiplicity of embryonic antigens during pregnancy. This is suggested by other studies (Baldwin and Embleton, 1974) where lymph node cells taken from rats bearing the essentially non-immunogenic mammary carcinomata employed in the present investigation were found to be cytotoxic *in vitro* for the autochthonous tumour and also other mammary carcinomata, but not histologically different tumour types. The neoantigens involved in these responses were viewed as being embryonic antigens since lymph node cell cytotoxicity could be blocked by treating target cells with multiparous rat serum. This, however, requires more direct evaluation since these conclusions are relevant to immunological studies of human malignant tissue where organ specific neoantigens have been identified (Hellström and Hellström, 1973).

This work was supported by a grant from the Cancer Research Campaign and by the award to one of us (B.M.V.) of a Medical Research Council Studentship.

REFERENCES

Alexander, P. (1972) Foetal Antigens in Cancer. *Nature, Lond.*, **235**, 137.

Baldwin, R. W. (1973) Immunological Aspects of Chemical Carcinogenesis. *Adv. Cancer Res.*, **18**, 1.

Baldwin, R. W. & Barker, C. R. (1967) Demonstration of Tumour-Specific Humoral Antibody against Aminoazo Dye-induced Rat Hepatomata. *Br. J. Cancer*, **21**, 793.

Baldwin, R. W. & Embleton, M. J. (1969*a*) Immunology of 2-Acetylaminofluorene-induced Rat Mammary Adenocarcinomas. *Int. J. Cancer*, **4**, 47.

Baldwin, R. W. & Embleton, M. J. (1969*b*) Immunology of Spontaneously Arising Rat Mammary Adenocarcinomas. *Int. J. Cancer*, **4**, 430.

Baldwin, R. W. & Embleton, M. J. (1971) Tumour Specific Antigens in 2-Acetylaminofluorene Induced Rat Hepatomas and Related Tumours. *Israel J. med. Sci.*, **7**, 144.

Baldwin, R. W. & Embleton, M. J. (1974) Neoantigens on Spontaneously Arising and Carcinogen Induced Rat Tumours Defined by *In vitro* Lymphocytotoxicity Assays. *Int. J. Cancer*, **13**, 433.

Baldwin, R. W., Embleton, M. J., Price, M. R. & Vose, B. M. (1974*a*) Embryonic Antigen Expression on Experimental Rat Tumours. *Transplantn Rev.*, **20**. In the press.

Baldwin, R. W., Glaves, D. & Pimm, M. V. (1971) Tumour-associated Antigens as Expressions of Chemically-induced Neoplasia and their Involvement in Tumor–Host Interactions. In *Progress in Immunology*. Ed. B. Amos. New York: Academic Press. p. 907.

Baldwin, R. W., Glaves, D., Pimm, M. V. & Vose, B. M. (1972*a*) Tumour Specific and Embryonic Antigen Expression on Chemically Induced Rat Tumours. *Ann. Inst. Pasteur*, **122**, 715.

Baldwin, R. W., Glaves, D. & Vose, B. M. (1972*b*) Embryonic Antigen Expression in Chemically Induced Rat Hepatomas and Sarcomas. *Int. J. Cancer*, **10**, 233.

Baldwin, R. W., Glaves, D. & Vose, B. M. (1974*b*) Differentiation between the Embryonic and Tumour Specific Antigens on Chemically Induced Rat Tumours. *Br. J. Cancer*, **29**, 1.

Burdick, J. F. & Wells, S. A. (1973) Cross-reactivity Between Cell-Surface Antigens on Different Murine Carcinogen-induced Tumors, Demonstrated by a Modified Isotopic Antiglobulin Test. *J. natn. Cancer Inst.*, **51**, 1149.

Deichman, G. I. (1969) Immunological Aspects of Carcinogenesis by Deoxyribonucleic Acid Tumor Viruses. *Adv. Cancer Res.*, **12**, 101.

Grant, J. P., Ladisch, S. & Wells, S. A. (1974) Immunological Similarities between Fetal Cell Antigens and Tumor Cell Antigens in Guinea-pigs. *Cancer, N.Y.* In the press.

Hellström, K. E. & Hellström, I. (1973) Lymphocyte Mediated Cytotoxicity and Blocking Serum Activity to Tumour Antigens. *Adv. Immunol.*, **8**, 209.

Laurence, D. J. R. & Neville, A. M. (1972) Foetal Antigens and their Role in Diagnosis and Clinical Management of Human Neoplasms: A Review. *Br. J. Cancer*, **26**, 335.

LE MEVEL, B. P. & WELLS, S. A. (1973) Foetal Antigens Cross-reactive with Tumour-specific Transplantation Antigens. *Nature, New Biol.*, **244**, 183.

MÉNARD, S., CALNAGHI, M. I. & DELLA PORTA, G. (1973) *In vitro* Demonstration of Tumor-specific Common Antigens and Embryonal Antigens in Murine Fibrosarcomas Induced by 7,12-dimethylbenz[*a*]anthracene. *Cancer Res.*, **33**, 478.

PASTERNAK, G. (1969) Antigens Induced by the Mouse Leukemia Viruses. *Adv. Cancer Res.*, **12**, 1.

THOMSON, D. M. P. & ALEXANDER, P. (1973) A Cross-reacting Embryonic Antigen in the Membrane of Rat Sarcoma Cells which is Immunogenic in the Syngeneic Host. *Br. J. Cancer*, **27**, 35.

WANG, M. (1968) Delayed Hypersensitivity to Extracts from Primary Sarcomata in the Autothonous Host. *Int. J. Cancer*, **3**, 483.